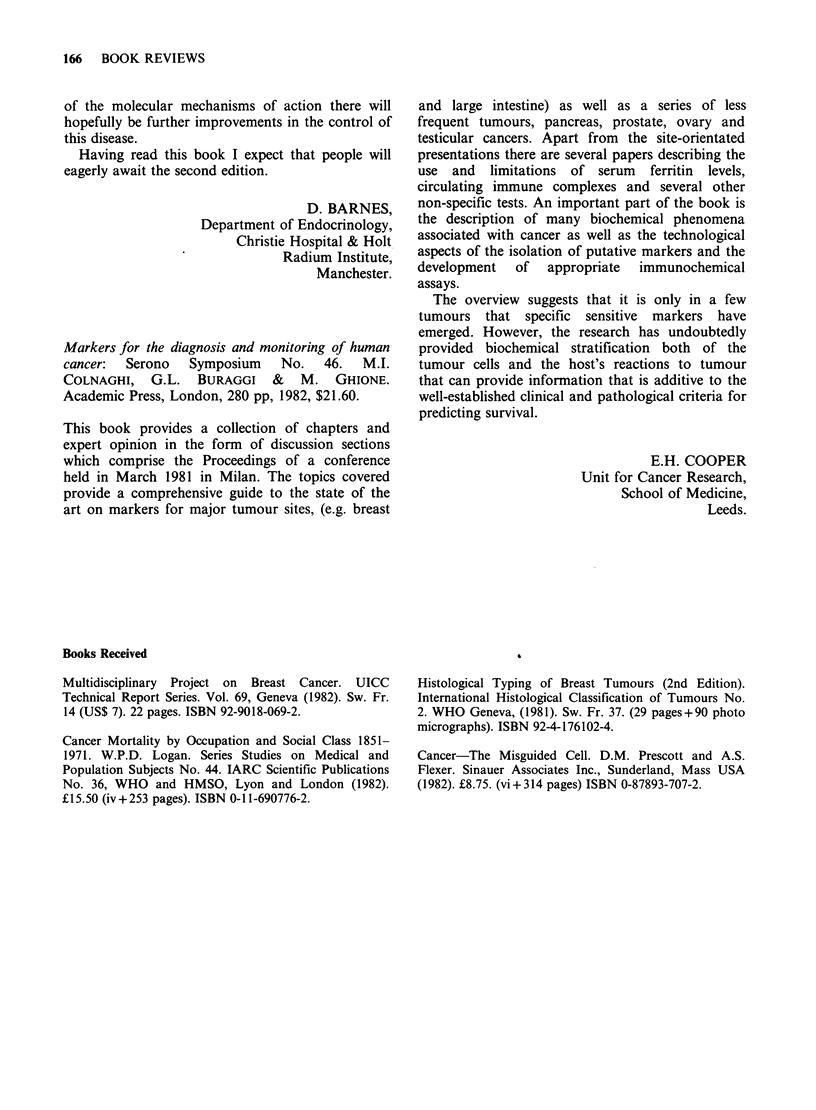# Markers for the diagnosis and monitoring of human cancer

**Published:** 1983-01

**Authors:** E.H. Cooper


*Markers for the diagnosis and monitoring of human cancer*: Serono Symposium No. 46. M.I. COLNAGHI, G.L. BURAGGI & M. GHIONE. Academic Press, London, 280 pp, 1982, $21.60.

This book provides a collection of chapters and expert opinion in the form of discussion sections which comprise the Proceedings of a conference held in March 1981 in Milan. The topics covered provide a comprehensive guide to the state of the art on markers for major tumour sites, (e.g. breast and large intestine) as well as a series of less frequent tumours, pancreas, prostate, ovary and testicular cancers. Apart from the site-orientated presentations there are several papers describing the use and limitations of serum ferritin levels, circulating immune complexes and several other non-specific tests. An important part of the book is the description of many biochemical phenomena associated with cancer as well as the technological aspects of the isolation of putative markers and the development of appropriate immunochemical assays.

The overview suggests that it is only in a few tumours that specific sensitive markers have emerged. However, the research has undoubtedly provided biochemical stratification both of the tumour cells and the host's reactions to tumour that can provide information that is additive to the well-established clinical and pathological criteria for predicting survival.

E.H. COOPER
Unit for Cancer Research,
School of Medicine,
Leeds.